# Femoral neck fracture in a senile patient with primary hyperparathyroidism: A case report

**DOI:** 10.1097/MD.0000000000040277

**Published:** 2024-11-22

**Authors:** Hong-Xia Zhu, Lei Fu, Yu Xie, Xiao Yuan, Sheng-Tao Chen, Lun-Li Xie

**Affiliations:** a Department of Traumatic Orthopedics, Hunan University of Medicine General Hospital, Huaihua, China; b Department of Joint and Hand Orthopedics, Hunan University of Medicine General Hospital, Huaihua, China.

**Keywords:** femoral neck fracture, hypercalcemia, parathyroidectomy, primary hyperparathyroidism

## Abstract

**Rationale:**

As a rare cause of femoral neck fracture, usually, hyperparathyroidism is missed diagnosed by orthopedist. Patient can present with various disappearance of clinical manifestations. Primary hyperparathyroidism in senile male population is commonly an asymptomatic disorder discovered incidentally through routine lab testing. Here, this study reports a case of femoral neck fracture in a senile patient with primary hyperparathyroidism.

**Patients concerns:**

A 70-year-old male patient with a known right femoral neck fracture associated with primary hyperparathyroidism.

**Diagnoses:**

A neck ultrasound (US) and computed tomography revealed a solid mass below the right lobe of the thyroid. X-ray plain and computed tomography confirmed right femoral neck fracture and multiple bone lesions. The routine lab testing showed hypercalcemia and hyperkalemia.

**Interventions:**

Before undergoing total hip arthroplasty surgery, patient was temporarily treated with hydration, diuretics, and calcitonin. Besides, the patient underwent parathyroidectomy of the enlarged parathyroid gland. Oral calcium preparations were routinely used for prevention of hypocalcemia.

**Outcomes:**

After completing all surgery, the patient was discharged without any complications including hypercalcemia and hyperkalemia.

**Lessons:**

Femoral neck fracture associated with primary hyperparathyroidism is a rare presentation. This case highlights that hypercalcemia and multiple osteopathy should be considered in the differential diagnosis in patients with pathological fracture caused by micro-traumatic injury.

## 1. Introduction

Senile patient with primary hyperparathyroidism (PHPT) has no obvious clinic symptom. Patient often is diagnosed with primary hyperparathyroidism because of occurring after incidental findings of hypercalcemia.^[1]^ The main clinical manifestations of PHPT are bone diseases with increased bone resorption such as pathological fracture, osteoporosis, and osteomalacia.^[2,3]^ Femoral neck fractures occurs commonly in patients with bone weakness due to osteoporosis or osteomalacia.^[3]^ Here we report the case of a senile male with right femoral neck fractures.

## 2. Case presentations

**Figure 1.**
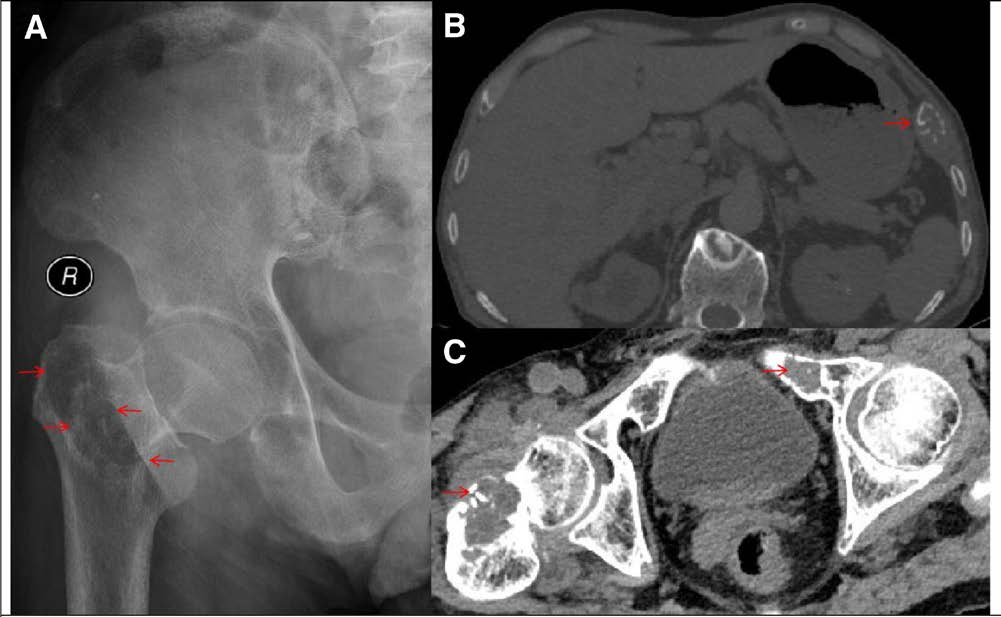
(A) The preoperative anteroposterior showed radiolucent appearance and femoral neck fracture (red arrows); (B) The pulmonary CT scan showed a obvious costal lytic lesion (red arrow); (C) The pelvic CT showed femoral neck fracture and upper branch of pubis with suspicious bone lesions (red arrows). CT = computed tomography.

**Figure 2.**
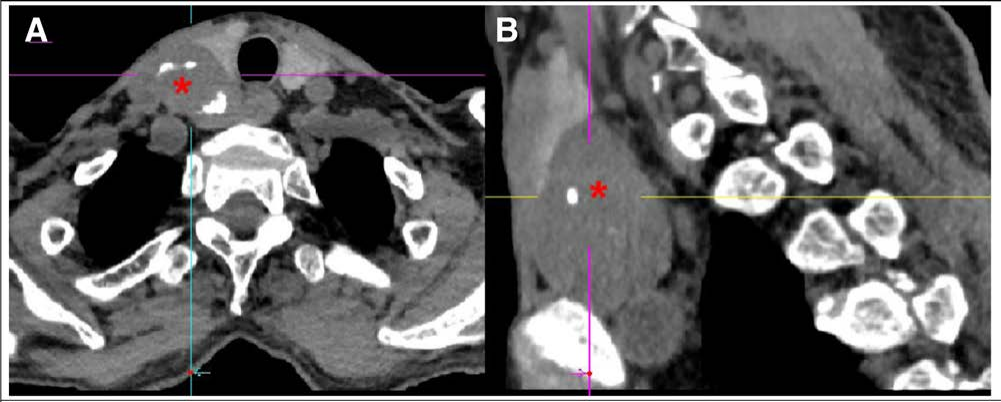
(A) and (B) The neck and pulmonary transversal and sagittal CT scan showed a solid mass below the right lobe of the thyroid (red asterisks). CT = computed tomography.

**Figure 3.**
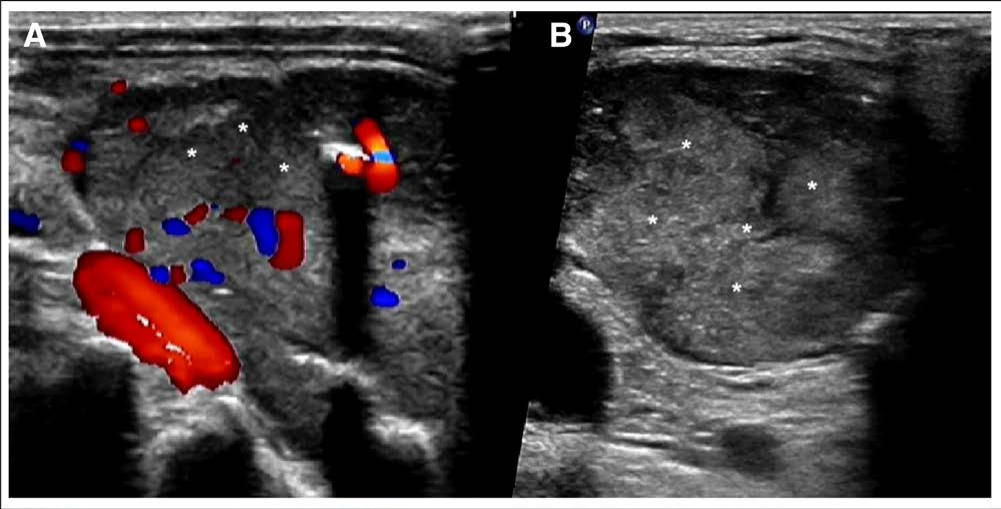
(A) and (B) The images of neck ultrasound showed this solid mass below the right lobe of the thyroid (White asterisks) with blood flow signals.

A 70-year-old male was presented to the emergency department with sudden occurrence of pain and restricted mobility in his right lower limb without any injury or muscle spasticity. He presented to an emergency department where X-ray films and computed tomography image were consistent with a right femoral neck fracture, upper branch of pubis, and costal bone lesion (Fig. 1). He had history of renal stone and pulmonary embolism. A solid mass below the right lobe of the thyroid was discovered incidentally through routine computed tomography test (Fig. 2). The patient was then immediately accepted neck ultrasound (US), and US confirmed the same solid mass below the right lobe of the thyroid (Fig. 3). Given that femoral neck pathological fracture caused by metabolic bone disease was suspected, blood work was drawn to aid in diagnosing its origin. The patient’s admission serum laboratory values were remarkable for severe serum hypercalcemia of 3.14 mmol/L (normal 2.07–2.65 mmol/L) and hyperkalemia of 5.76 mmol/L (normal 3.5–5.3 mmol/L). He was also found to have a low serum concentration of vitamin D 25-hydroxy at 14 ng/mL (normal between 30 and 100 ng/mL). Primary hyperparathyroidism was then definitively diagnosed with an elevated serum parathyroid hormone of 94.8 pmol/L (normal 1.96–9.33 pmol/L).

**Figure 4.**
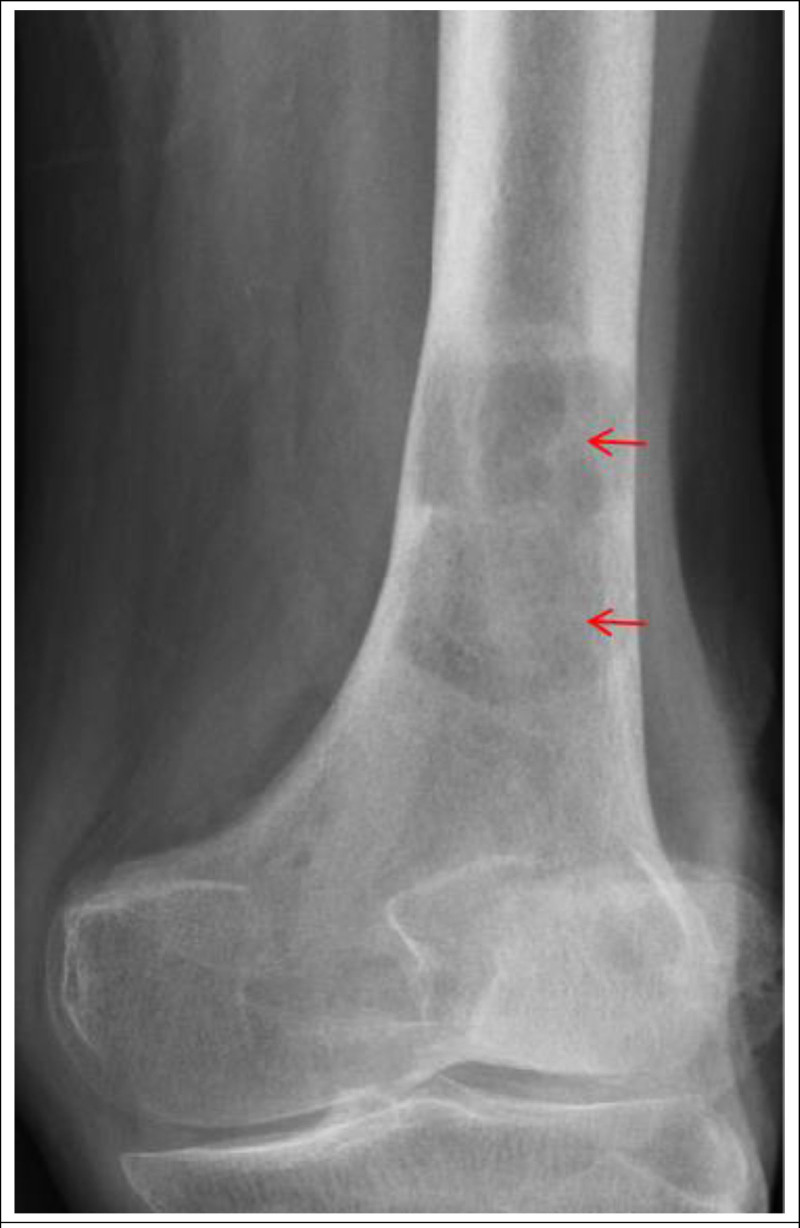
Radiographs of the right distal femur was also taken and showed bone lytic lesions (red arrows).

**Figure 5.**
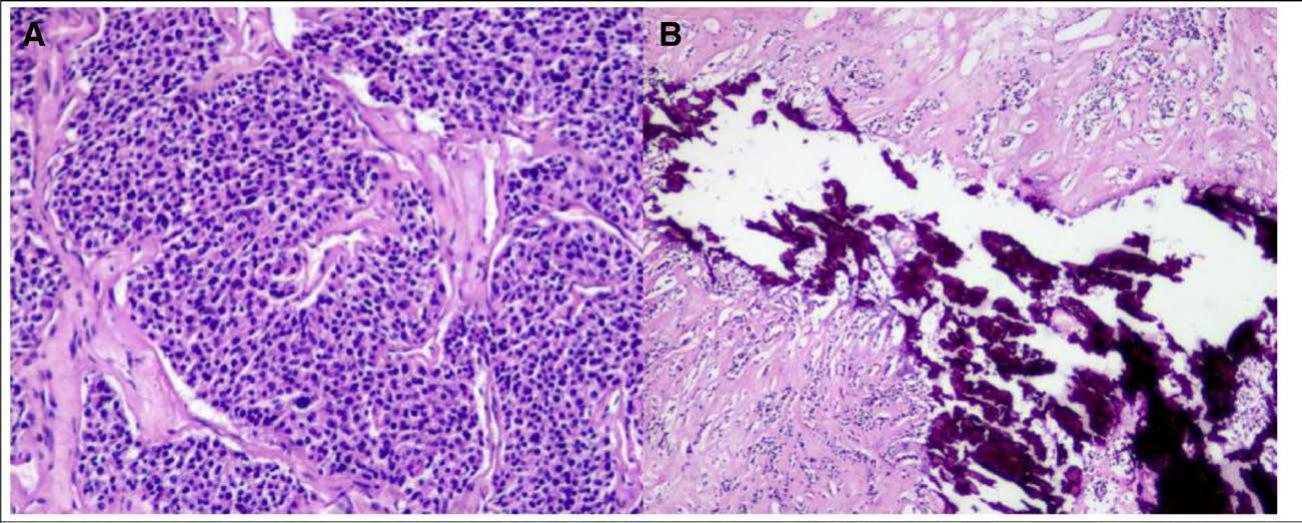
(A) and (B) Pathological findings revealed atypical hyperplasia of parathyroid adenoma cell s(Hematoxylin and Eosin staining, magnification 40), and immunohistochemistry showed Syn(+), CgA(+), TTF-1(−), Cyclindl(+), TG(−), Galectin-3(+), Ki-67(15%+).

Considering downstream consequences of hyperparathyroidism, several other medical inspection were obtained. Bilateral renal ultrasounds showed renal stones, without adrenal gland masses. Radiographs of the right distal femur was also taken and showed bone lytic lesions (Fig. 4). His hyperkalemia were believed to be related to hypercalcemia. Hydration, diuretics, and calcitonin were temporarily used to treatment of his hypercalcemia. After that, he underwent unilateral parathyroidectomy of parathyroid gland on hospitalization duration. Preliminary pathology revealed this was an atypical hyperplasia of parathyroid adenoma (Fig. 5). Following parathyroidectomy, the patient accepted therapy of effective electrolyte management. As expected, he developed rebound hypocalcemia due to hungry bone syndrome, requiring routine calcium supplementation by oral calcium preparations combined with intravenous calcium chloride. Notably, serum potassium was normal level in his repeated detection of electrolyte. His serum parathyroid hormone decreased to 1.8 pmol/L (normal 1.96–9.33 pmol/L).

**Figure 6.**
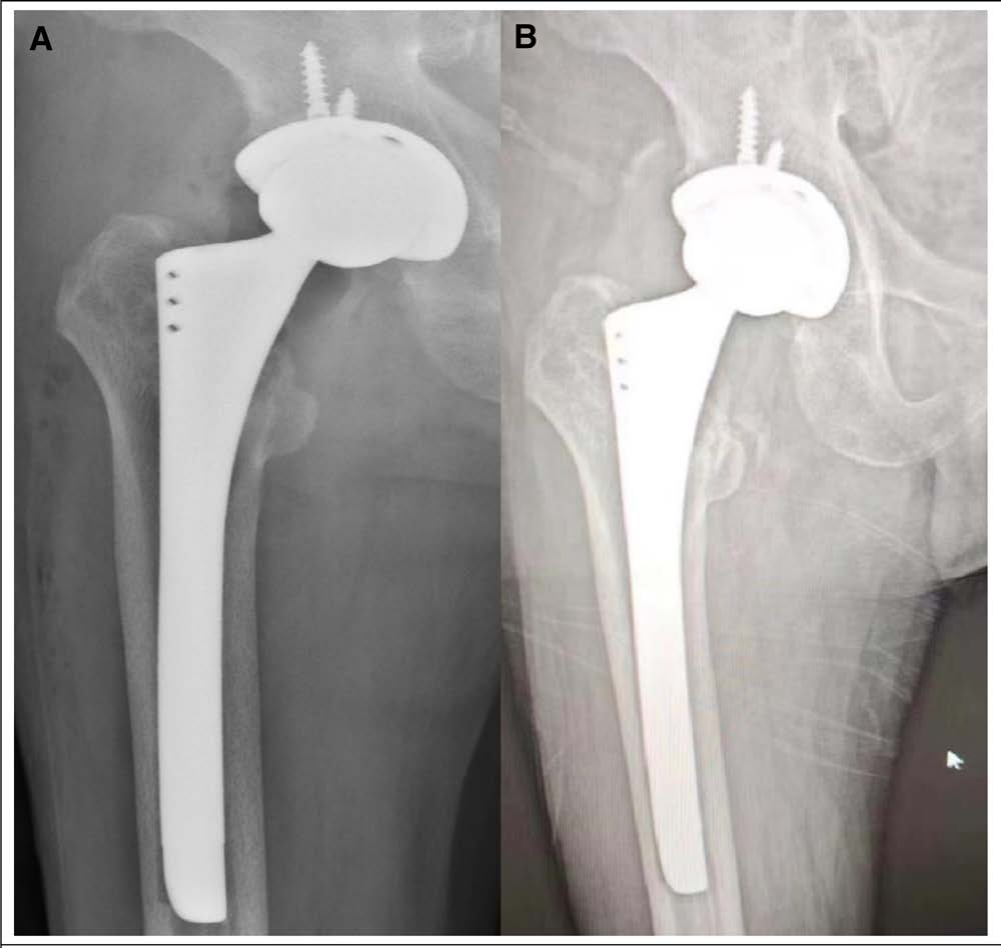
(A) The postoperative anteroposterior X-ray showed total hip arthroplasty for right femoral neck fracture. (B) Follow-up X-ray of hip joint demonstrated stable conditions of bone and hip prosthesis.

**Figure 7.**
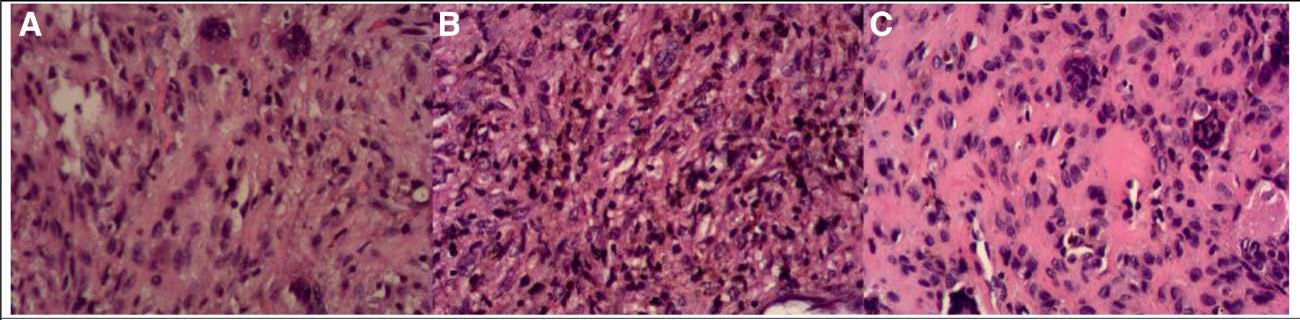
(A–C) Pathological findings revealed osteoplastica of femoral neck or osteitis fibrosa cystica, femoral head necrosis along with proliferation of fibrous tissue in the medullary cavity and infiltration of inflammatory cell (Hematoxylin and Eosin staining, magnification 40).

When he acquired stable levels of serum electrolytes, he underwent total cementless hip arthroplasty (Chun-Li BE Type, Beijing, China) for right femoral neck fracture (Fig. 6). Provided by Beijing Chun-Li Zhengda Medical Equipment Co., LTD., Certificate No.20173134472, Medical Equipment Production License No. (Beijing) 20000395. Postoperative pathological examination showed osteoplastica of femoral neck, femoral head necrosis along with proliferation of fibrous tissue in the medullary cavity (Fig. 7). The patient’s recovery was uncomplicated, he was kept on walking aids after first postoperative week. Three-months telephone and micro-letters follow-up proved that he had stable conditions (Fig. 6). He was not seen again despite a concerted effort by the medical team for additional follow-up.

## 3. Discussion

As the causes of pathological fracture, primary hyperparathyroidism is a rare disorder comparing with osteoporosis particularly in older population. Previous studies reported that the incidence rate of PHPT varies from 34 to 120 cases per 100,000 individuals, and the prevalence of PHPT in postmenopausal women is >2%.^[4,5]^ Because PHPT is relatively rare in the clinical setting and some clinicians have an insufficient understanding of this disease, in particular, orthopedic surgeons fail to make a timely and correct diagnosis, and are prone to misdiagnosis.^[5]^ Most patients with primary hyperparathyroidism are first diagnosed in the department of orthopedics because of pathological fractures as main manifestation.^[6]^ Thus, this an unusual presentation often results in a high rate of delayed treatment or misdiagnosis.^[7]^ Besides, it may highlight teachable points for orthopedic surgeons. Our case report a senile patient with pathological fracture and multiple bone lesions secondary to primary hyperparathyroidism, who was first misdiagnosed as metastatic bone tumors.

PHPT is caused by abnormal elevation of parathormone due to parathyroid gland lesions including parathyroid adenoma, parathyroid hyperplasia, and parathyroid carcinoma, all of which are mainly managed by surgical treatment.^[5]^ In ordinary medical inspection, no obvious clinic symptoms are usually discovered by clinician.^[8]^ Nevertheless, some patients have nonspecific symptoms such as osteoporosis.^[5,9]^ Moreover, very few patients have specific manifestations such as bone pain, pathological fracture, or other bone diseases.^[5]^

As a types of endocrine disorders, early PHPT often brings a series of secondary atypical symptoms and signs by mainly acting on the skeletal system and urinary system.^[10]^ Actually, PHPT are certainly proved to effect on other systems such as the digestive system, nervous system, and muscular system.^[10,11]^ In our case, osteoporosis, multiple bone lesions, renal stones, hypercalcemia and hyperkalemia was diagnosed by radiographic examination and routine lab testing. The study suggested that these symptoms are caused by parathormone-induced enhancement of the effect of osteoclasts, which causes calcium in bones to be dissolved into the blood, resulting in hypercalcemia causing causing urinary calculi or renal insufficiency, when the concentration of calcium ions exceeds the renal threshold.^[5]^ And then, hyperkalemia occurs with the deterioration of renal function.^[5]^

Methods of treatment for PHPT involves resection or removal of the source of excessive parathyroid hormone, as well as treating pathology stemming from the hypercalcemic state.^[12]^ Parathyroidectomy is the treatment of choice for senile patient with PHPT. However, as a common complication, hypocalcemia is divided it into different types: postoperative hypoparathyroidism, protracted hypoparathyroidism, and permanent hypoparathyroidism.^[13]^ On perioperative duration, our case accepted routine calcium supplementation by oral calcium preparations combined with intravenous calcium chloride.

Our case reported a 70-year-old male patient who were capable of independent walking before the injury. Thus, he underwent total biotype hip arthroplasty with lengthened single-modular femoral stem according to the scoring system for patients with femoral neck fracture.^[14]^ The following reasons were considered by us. Firstly, basing on individual conditions with proximal femoral bone destruction and his age, in our case, the lengthened single-modular femoral stem was used to prevention of prosthesis subsidence or loosening at long-term stage.^[15]^ Secondly, comparing to dual-modular femoral stems, a single-modular femoral stem has been proved by study, which showed better long-term results after total hip arthroplasty.^[15,16]^ Thirdly, to avoid occurrence of postoperative acetabular pain caused by artificial femoral head, surgery strategy was total hip arthroplasty.^[17]^ Finally, to precaution of total hip arthroplasty dislocation, some measures include avoiding to usage small diameter of artificial femoral head(<30 mm), suitable operative approach, appropriate position of prosthetic component, and postoperative body position control.^[18,19]^

## 4. Conclusion

In conclusion, we have herein reported a rare case of pathological femoral neck fracture secondary to PHPT. Through reflection of the whole process of diagnosis and treatment of this case, we conclude that osteoporosis is not the only cause of femoral neck fracture in senile patient, in particular, who associated with abnormalities of serum electrolyte. Some comprehensive diagnostic methods need to exclude multiple bone destruction caused by PHPT. Our findings showed that a clear diagnosis and surgical resection of the parathyroid gland as early as possible can yield good therapeutic results for patients with femoral neck fracture associated with PHPT. Clinicians should consider the possibility of bone metastases or endocrine correlative disorders in elder patients with pathological fracture except that osteoporosis.

## Author contributions

**Conceptualization:** Hong-Xia Zhu, Lun-Li Xie.

**Data curation:** Hong-Xia Zhu, Lei Fu, Yu Xie.

**Formal analysis:** Xiao Yuan, Sheng-Tao Chen.

**Funding acquisition:** Lun-Li Xie.

**Investigation:** Hong-Xia Zhu, Lun-Li Xie.

**Methodology:** Xiao Yuan, Sheng-Tao Chen.

**Project administration:** Lun-Li Xie.

**Visualization:** Hong-Xia Zhu, Lun-Li Xie.

**Writing – original draft:** Hong-Xia Zhu, Lei Fu.

**Writing – review & editing:** Lun-Li Xie.
